# Diagnosis of *SLC25A46*-related pontocerebellar hypoplasia in two siblings with fulminant neonatal course: role of postmortem CT and whole genomic analysis: a case report

**DOI:** 10.1186/s12883-021-02540-x

**Published:** 2022-01-10

**Authors:** Mamiko Yamada, Hisato Suzuki, Hiroyuki Adachi, Atsuko Noguchi, Fuyuki Miya, Tsutomu Takahashi, Kenjiro Kosaki

**Affiliations:** 1 Center for Medical Genetics, Keio University School of Medicine, Tokyo, Japan; 2 Center for Medical Genetics, Keio University School of Medicine, Tokyo, Japan; 3 Department of Pediatrics, Akita University Graduate School of Medicine, Akita, Japan; 4 Department of Pediatrics, Akita University Graduate School of Medicine, Akita, Japan; 5 Center for Medical Genetics, Keio University School of Medicine, Tokyo, Japan; 6 Department of Pediatrics, Akita University Graduate School of Medicine, Akita, Japan; 7 Center for Medical Genetics, Keio University School of Medicine, Tokyo, Japan

**Keywords:** Pontocerebellar hypoplasia, *SLC25A46*, Genomic analysis, Whole genome sequencing

## Abstract

**Background:**

Pontocerebellar hypoplasia (PCH) is increasingly known as a degenerative disease rather than simple “hypoplasia”. At least 21 disease-causing genes have been identified for PCH so far. Because PCH is very heterogenous, prognostic prediction based solely on clinical or radiologic findings is not feasible.

**Case presentation:**

Here, we report two siblings who had a fulminant neonatal course. The documentation of pontocerebellar hypoplasia by postmortem brain CT imaging in one of the siblings and a subsequent complex and comprehensive whole genome analysis established that both siblings had bi-allelic compound heterozygous variants (a splicing variant and a deletion) in the *SLC25A46* gene which encodes a solute carrier protein essential for mitochondrial function. Long-read whole genome sequencing was required to confirm the presence of the deletion. The fulminant courses suggest that *SLC25A46*-related PCH is an acutely progressive degenerative condition starting in utero, rather than a simple static hypoplasia.

**Conclusion:**

The genomic analysis was instrumental and essential to solving the enigma of the unexplained neonatal deaths of these two siblings and to provide accurate genetic counseling.

## Background

Pontocerebellar hypoplasia (PCH) is characterized by the degeneration of the cerebellum and pons and was first described in 1917. Other features include severe psychomotor retardation, optic nerve atrophy, progressive myoclonic ataxia, and axonal peripheral neuropathy [1]. Indeed, “Hypoplasia” is a misnomer because the loss of volume in the cerebellum and pons is caused by the degeneration of Purkinje cells, rather than by true developmental hypoplasia [2]. Although there are no indications for surgical interventions for PCH, recognition of this progressive entity is critical because the natural course is completely different from those of other non-progressive disorders.

PCH has been categorized into several subtypes. So far, at least 21 disease-causing genes are known [3, 4]. One such causative gene, *SLC25A46* encodes solute carrier family 25 member 46, a modified carrier protein that is recruited to the mitochondrial outer membrane and is involved in the maintenance of mitochondrial cristae structure by interacting with mitofusin2 (MFN2), Optic Atrophy 1 (OPA1), and the mitochondrial contact site and cristae organizing system [5]. So far, *SLC25A46* variants have been reported in 19 PCH patients from 14 unrelated families [6, 7].

Here, we report two siblings with *SLC25A46* variants who had fulminant and lethal neonatal courses. Genomic diagnosis was achieved through the results of sophisticated whole genome sequencing methods, which correlated with post-mortem brain CT.

## Case presentation

**Fig. 1:**
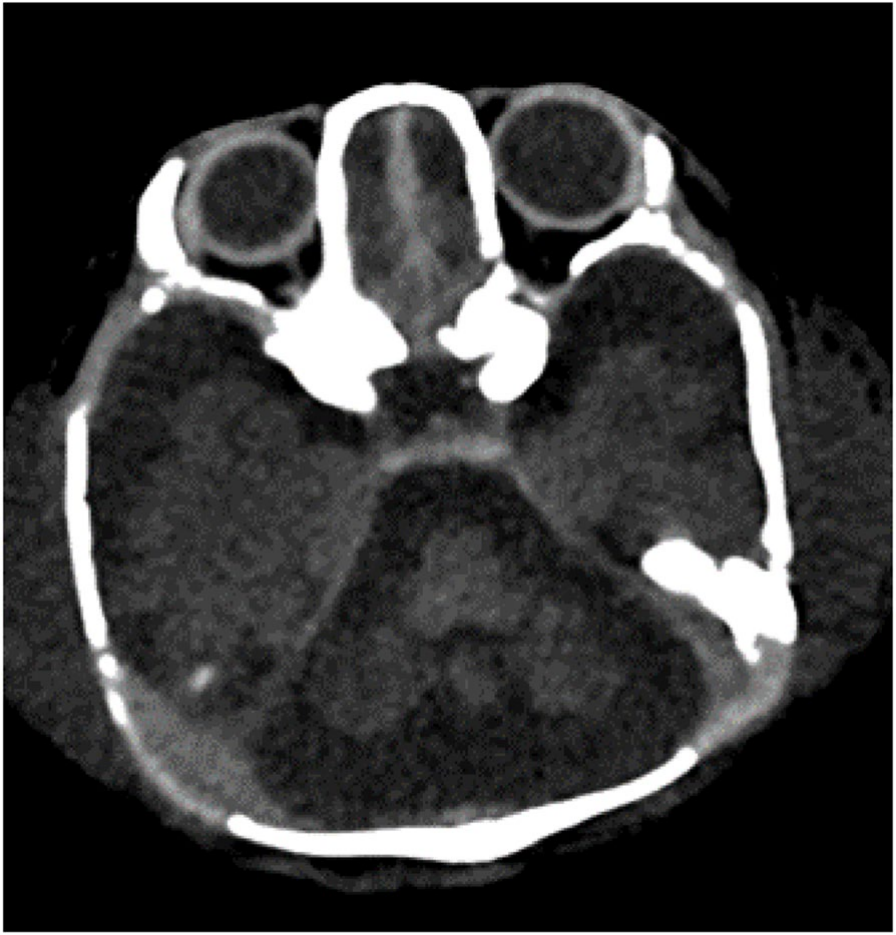
Postmortem head CT imaging of the patient. The CT imaging showed hypoplasia/atrophy of the cerebellum and enlarged cisterna magna

The proband was a 15-day-old male born at 37 weeks and 1/7 days of gestation to unrelated parents. His birthweight was 2578 g (− 0.16 SD), his body length was 47 cm (− 0.22 SD), and his head circumference was 35.3 cm (1.92 SD). The pregnancy was a spontaneous one, and amniotic fluid overload, fetal effusion, and fetal ascites were noted after 36 weeks of pregnancy. The patient’s Apgar Score was severely depressed (i.e., 1 at 1 min, 1 at 5 min, and 2 at 10 min, with all scores for pulse only), and all other parameters such as appearance, grimace, activity and respiration were zero [8]. At birth, the baby was not breathing spontaneously and did not respond to resuscitation, resulting in severe neonatal hypoxic-ischemic injury. Further treatment included cardiovascular support, inhaled nitric oxide therapy, and high frequency oscillatory ventilation, but the response to any treatment was poor, with worsening edema, increased pleural effusions, ascites, and anuria. The pleural fluid was not chyle, and lymphatic hypoplasia was ruled out. The lungs of the baby were hypoplastic. Because of the patient’s critical condition, imaging evaluation of the brain could not be performed, but trans fontanel ultrasonography showed an enlarged cisterna magna. The patient died at the age of 19 days. Postmortem CT imaging of the head showed cerebellar hypoplasia (Fig. 1). No autopsy was performed.

A significant family history included an elder brother who had died at the age of 12 h. The brother had been born at 36 weeks and 6 days of gestation and had been noted to have a breech presentation and polyhydramnios during the fetal period; he passed away because of severe neonatal asphyxia and cardiopulmonary failure with hypoplastic lungs. The parents had no diseases of note.

**Fig. 2:**
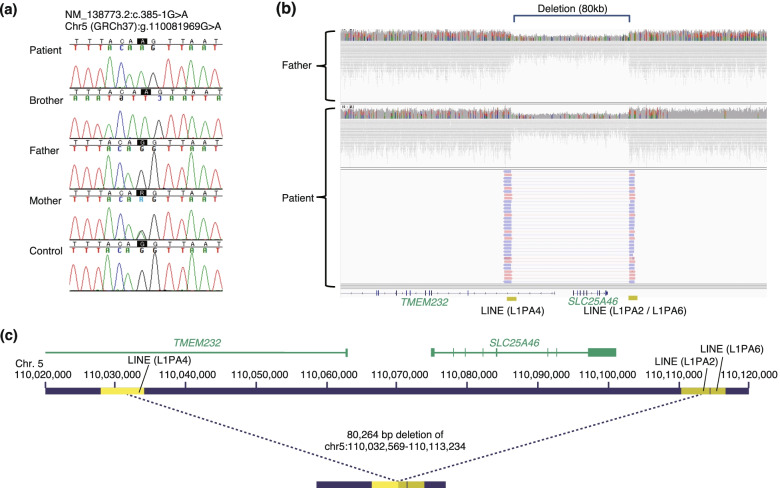
Molecular analysis of the patient and his family members. (a) A Sanger sequencing of the DNA derived from the peripheral blood of the proband and his parents and from dried umbilical cord of his deceased brother revealed that the patient and his deceased brother had a hemizygous variant of the *SLC25A46* gene, Chr5(GRCh37):g.110081969G > A, NM_138773.2 c.385-1G > A, which was derived from his mother. (b) Paternally derived 80-kb deletion spanning the *TMEM232* and *SLC25A46* loci. Top: Coverage diagram of the short-read whole genome sequencing in the father. Bottom: Coverage diagram of the short-read whole genome sequencing and long-read sequencing of PCR products amplified across the deletion. The deletion spans highly homologous L1 elements (L1PA4 in the intron of the*TMEM232* locus and L1PA2 at the intergenic region telomeric to the *SLC25A46*). (c) Detailed view of the extent of the deleted region based on GRCh37 (hg19) coordinates

An exome analysis from the peripheral blood of the proband and his parents was performed, as previously reported [9]. The proband had an apparently homozygous variant of the *SLC25A46* gene (Chr5(GRCh37):g.110081969G > A, NM_138773.2 c.385-1G > A) (Fig. 2a). This variant is located within the canonical consensus “AG” dinucleotide. The predicted effect of this variant on splicing was high (0.8 within the 0–1.0 scale) according to the deep learning-based tool SpliceAI [10]. As for the deceased brother, the dried umbilical cord had been preserved because for religious reasons. Targeted sequencing was performed on the cord-derived DNA, and an apparently homozygous variant identical to that of the patient was detected. The mother was heterozygous for the c.385-1G > A allele, but the father did not have this variant. We considered two possibilities: uniparental isodisomy, or a heterozygous deletion derived from the father. Uniparental isodisomy was less likely based on the recurrence of “apparent homozygosity” in the two affected siblings. Furthermore, homozygosity mapping of the exome data using the computer program H3M2 excluded uniparental disomy [11].

Multiple programs for the detection of copy number variants from exome data, including XHMM, Excavator2, and FishingCNV were used to search for a potential deletion involving the *SLC25A46* locus [12–14]. None of these programs detected a deletion spanning the *SLC25A46* locus. Whole genome sequencing of the proband’s sample was performed using a short-read sequencer (NovaSeq6000; Illumina) according to the manufacturer’s instructions. Samples were processed through an alignment and structural variant detection pipeline using the DRAGEN 3.5 suite for the Illumina data. The presence of an 80-kb deletion (chr5:110,032,569-110,113,234) (GRCh37/hg19) spanning *SLC25A46* and *TMEM232* was detected in both patients and the father (Fig. 2b and c). *TMEM232* is not known to be associated with human diseases.

To better characterize the breakpoint, we designed polymerase chain reaction (PCR) primers for the amplification of genomic regions spanning the breakpoints. The left primer was 5′-CCCGAGCTATTAGTCCTCAAAC-3′, and the right primer was 5′-AATGATTTTGCCCCATCATTAC-3. The amplicon was further analyzed using a long-read sequencer (PromethION system; Oxford Nanopore Technologies). Briefly, a sequencing library was constructed from the amplicon using the Ligation Sequencing Kit SQK-LSK109 and the Native barcoding Expansion Kit EXP-NBD104, following the manufacturer’s instructions; sequencing was then performed using the FLO-PRO002 R9.4.1 Flow Cell on the PromethION system. Basecalling and demultiplexing were performed using Guppy Basecaller v.4.3 on the same instrument. The obtained long read sequences were mapped against the GRCh37/hg19 human reference genome using the LRA program v1.1.2 [15], and the breakpoint of the deletion was determined by manual inspection using the Integrative Genomics Viewer [16]. Both breakpoints were within highly homologous LINE transposon sequences, L1PA4 and L1PA2. The deletion was likely to be mediated through non-allelic homologous recombination (Fig. 2c).

## DISCUSSION and CONCLUSIONS

Here, we report two siblings with PCH caused by bi-allelic compound heterozygous variants in the *SLC25A46* gene. A genomic analysis was instrumental and essential to solving the enigma of the unexplained neonatal deaths of the two siblings. The fulminant courses indicate that *SLC25A46*-related PCH is as an acutely progressive degenerative condition starting in utero, rather than a simple static hypoplasia.

Our observation gives further credence to the emerging notion that biallelic loss-of-function variants (e.g., truncating and splicing variants or gene deletion) of *SLC25A46* lead to PCH. The fulminant neonatal course of PCH is in sharp contrast to other phenotypic expressions of biallelic variants of *SLC25A46*, including “Neuropathy, hereditary motor and sensory, type VIB (OMIM: 616505),” optic atrophy and parkinsonism.

The concept of PCH was first coined by Dr. Peter G. Barth [17] as a degenerative brain disorder with a fetal onset. Later it became apparent that some types of PCH are progressive whereas others are non-progressive. Furthermore, a large number of disorders have imaging patterns that are compatible with PCH (i.e., reduced volumes in the pons and cerebellum) [18, 19]. Hence, a genomic etiologic investigation is desirable, in addition to radiologic studies in the evaluation of reduced pons and cerebellum volumes because both the prognosis and recurrence risk depend on the etiologic diagnosis.

The identification of both mutant alleles was crucial in providing a precise genetic diagnosis and subsequent genetic counseling, including information on possible preimplantation diagnosis. A conventional exome analysis using short-read sequencing succeeded in the detection of one mutant allele, but not the other mutant allele. The utilization of long-read whole genome sequencing resulted in the detection of the deletion and its extent, ultimately leading to a confirmatory diagnosis. The diagnostic clue for finding the deletion in the presently reported family was “apparent homozygosity” for the splice-disrupting allele detected using short-read sequencing. The lack of the allele in one of the parents (i.e., the father) despite “apparent homozygosity” prompted a further analysis using short-read whole genome sequencing and subsequent long-read whole genome sequencing. The serial application of increasingly sophisticated analytic methods led to the eventual success in the detection of the deletion derived from the father.

In view of recent studies showing the central role of mitochondrial defects in degenerative forms of PCH, various drugs used for mitochondrial protection may be helpful for patients with *SLC25A46* defects [20]. A prompt and precise definition of the causative gene in each patient with PCH during the neonatal period may open a door to potential pharmacologic interventions in the future.

## Abbreviations

PCH: PontoCerebellar hypoplasia
*SLC25A46*: Solute Carrier Family 25 Member 46
MFN2: Mitofusin2
OPA1: Optic Atrophy 1
